# An Intelligent Decision-Making Support System for the Detection and Staging of Prostate Cancer in Developing Countries

**DOI:** 10.1155/2020/5363549

**Published:** 2020-08-17

**Authors:** Jun Zhang, Zhigang Chen, Jia Wu, Kanghuai Liu

**Affiliations:** ^1^School of Computer Science and Engineering, Central South University, Changsha 410083, China; ^2^Central South University, Changsha 410075, China

## Abstract

Most developing countries face huge challenges in the medical field; scarce medical resources and inadequate medical personnel will affect the development and stability of the society. Therefore, for most developing countries, the development of intelligent medical systems can greatly alleviate the social contradictions arising from this problem. In this study, a new data decision-making intelligent system for prostate cancer based on perceptron neural network is proposed, which mainly makes decisions by associating some relevant disease indicators and combining them with medical images. Through data collection, analysis and integration of medical data, as well as the disease detection and decision-making process, patients are given an auxiliary diagnosis and treatment, so as to solve the problems and social contradictions faced by most developing countries. Through the study of hospitalization information of more than 8,000 prostate patients in three hospitals, about 2,156,528 data items were collected and compiled for experiment purposes. Experimental data shows that when the patient base increases from 200 to 8,000, the accuracy of the machine-assisted diagnostic system will increase from 61% to 87%, and the doctor's diagnosis rate will be reduced to 81%. From the study, it is concluded that when the patient base reaches a certain number, the diagnostic accuracy of the machine-assisted diagnosis system will exceed the doctor's expertise. Therefore, intelligent systems can help doctors and medical experts treat patients more effectively.

## 1. Introduction

Prostate cancer (PCA) is a human disease that occurs in malignant tumors of the prostate epithelium [1–6]. The age of its onset is mainly after 55 years of age, and the incidence rate also increases with the increase of age. In Europe and the United States, the incidence of male cancer patients ranked first, [7, 8] while the death rate ranked second. In America, the incidence of prostate cancer has risen to become the first place; the mortality rate is second only to lung cancer. Prostate cancer has become one of the most common cancers in the world and one of the most common malignancies among men in Europe and the United States [9].

In developing countries such as [10] China, the incidence rate is lower than that in many European and American countries. However, due to the large population base in developing countries, the number of cases is not to be underestimated [11]. Before the 1980s, the incidence of prostate cancer in China was less than 1/100,000, and the incidence was extremely low, but after decades of change, the incidence of prostate cancer has now exceeded 5/100,000, and it is skyrocketing, nearly fivefold to the original. Specifically, the incidence of prostate cancer registered in China in 2012 was 9.92/100,000; the sixth highest incidence of malignant neoplasms in men, and in 2018, prostate cancer patients in Asia accounted for 18,100,001 and half of the world's new patients [12, 13].

Nowadays, many developing countries are facing big dilemmas:

- Medical equipment and treatment level are limited, and with the increase of cancer incidence, it is very easy to cause high misdiagnosis rate if the medical level cannot keep up with the rate of growth of morbidity. Therefore, a large number of social contradictions may appear
- The large population base in developing countries and the relatively small number of medical institutions, as well as medical personnel, have led to an imbalance in the number of doctors and patients

The capital of China, Beijing, has a large population and has nearly 100,000 old people and children. But on the contrary, there are only 3,000 medical staff serving people in this metropolis. As a result, there is an extreme imbalance between the size of the population and the level of medical. In developing countries, even the big cities still have so many problems, let alone small cities in remote areas. It is usually more acute that the ratio of urban to rural population is about 4 : 6 in 2017 [14], but the city's medical level is much higher than that in the rural.

In developing countries, doctors in hospitals have to bear a huge burden because of heavy work and mental stress. In China, due to the huge population base, each doctor is allocated to 5,000 patients on average, while a lot of work pressure often leads to high rate of misdiagnosis. Moreover, [15, 16] in the treatment of prostate cancer, 640 images are generated for each patient by PET-CT scan. If the screening test is performed only by manual means, it will be very inefficient and with mental fatigue, working time is too long, and the rate of misdiagnosis will also increase.

Therefore, hospitals in developing countries have similar dilemmas:

- A large number of patients and relatively few medical staff form sharp contradictions, which leads to a large workload of medical staff, easy to cause physical fatigue and mental pressure of doctors, and eventually may increase the rate of misdiagnosis
- For example, screening PET-CT [17] image is a task with a large number of images and a large amount of repetitive work. If handled manually, it will only be inefficient, waste a lot of time, but also will fatigue the doctor easily, and, last, resulting in a high rate of misdiagnosis
- There is a “generation gap” between doctors and patients. Patients do not understand the physiological indicators of prostate cancer, which makes it difficult for doctors to communicate with patients

Faced with these problems, the article uses an intelligent medical expert system to improve the above medical conditions. The doctor's final choice can be combined with his own experience through system-assisted diagnosis to make the most complete decision-making treatment. In this system, a neural network-based prostate cancer big data intelligent system is adopted. The system assists the diagnosis decision from two aspects: image and disease index. By adjusting the weights of the two, the two aspects are finally combined to determine the clinical stage of cancer and provide appropriate treatment strategies for physicians. Big data decision-making intelligence system can complete the phased decision of disease quickly and effectively and at the same time can provide timely and effective treatment adjustment to the patient to ensure effective treatment of the disease. In summary, the contributions made by the research are summarized as follows:

1. To construct a new model of prostate cancer based on perceptron neural network, which combines a series of indicators and related images to determine whether there is cancer and to determine the severity of the cancer, up to the clinical stage
2. According to the images obtained by positron emission computed tomography (PET) or computed tomography (CT) taken by the hospital technicians [18–21], the images can be screened, and the most effective part of the images can be screened for doctors' reference, so as to reduce the burden of doctors and improve work efficiency
3. The intelligent system assists in medical diagnosis and through statistical analysis data can be used as an auxiliary diagnosis and automatically provide a fast and accurate treatment plan for doctors
4. In order to ensure that the learning neural network can obtain accurate weights and ensure the accuracy of decision-making, the research is based on 2,156,528 hospitalization information of three hospitals in China. The experimental results show that the intelligent medical decision-making system can improve the efficiency of doctors and reduce the rate of misdiagnosis

The rest of this paper is organized as follows: in the second part, introducing the current state-of-the-art related to our work; the third section proposes and constructs a big data decision model; the fourth section gives a detailed description of the experimental performance; the conclusion is given in the last part.

## 2. Material and Methods

The intelligent auxiliary diagnosis system is a new type of intelligent system based on machine learning and its application has been recognized by the society to a large extent. Through a large number of precedents, the intelligent system can still greatly facilitate people's lives and reduce the related work and life burden. The emergence of the intelligent medical system is expected to improve the current medical and health conditions in developing countries. With the update and development of technology, in many countries and regions, the intelligent medical system can assist in diagnosing of the diagnosis of the patient's health care system information of patients and to help doctors to do some simple relative work, which greatly reduces the workload of doctors, and even able to diagnose the illness and make corresponding treatment decisions, making doctors' to have a more effective understanding of the health situation. On the other hand, in order to increase the accuracy and rationality of machine-assisted diagnosis, the article should not only use simple indicators to make the decision process but also include more important PET-CT images to make the decision, because sometimes, the images can judge the disease more directly, accurately, and effectively. Therefore, the article establishes a new decision model based on the perceptron neural networks in developing countries.

### 2.1. General Framework of the Machine-Aided Diagnosis System Model of Prostate Cancer in the Context of Big Data

During the real medical diagnosis of prostate cancer, doctor makes the corresponding diagnosis through a variety of indicators such as PAP, PSMA, HB, PSMA, TPSA, RBC, PAP, FPSA and patients of positron emission computed tomography (PET) and computed tomography (CT). In real life, doctors will diagnose and treat according to their own knowledge and treatment experience.

**Figure 1:**
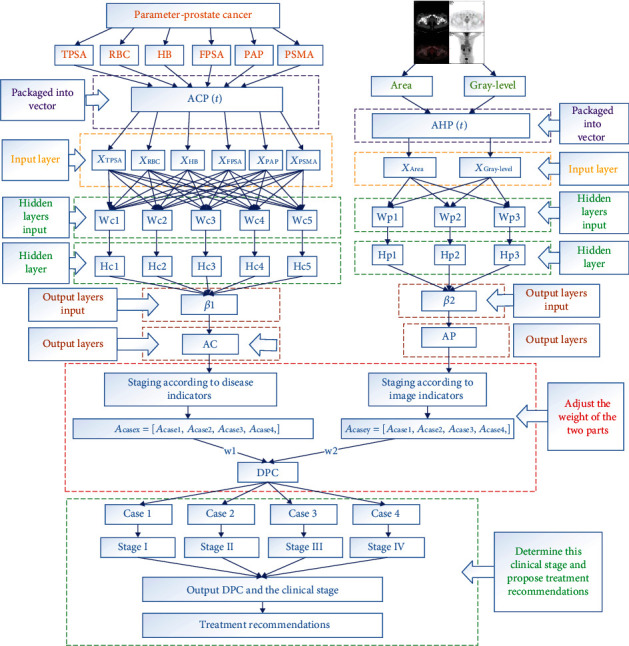
The overall structure of the big data-assisted diagnostic decision model.

In a big data environment, our medically assisted diagnostic systems are also based on big data. Through machine learning, a large amount of data is learned and a mature neural network algorithm is constructed to perform machine diagnosis through medical data and image information. As shown in Figure 1, it is the overall structure of the big data decision model of the intelligent medical system, and it introduces the system's operation process and data flow and processing in detail and clearly.

Finally, because of the strong concurrency of the hospital's intelligent system, it is able to process a large number of patient information at the same time. The system is mainly divided into five stages. The first stage is the preprocessing model of medical images before the input of the machine-aided diagnosis system of prostate cancer under the big data environment. Finally, it achieved two goals. One is accurate and effective screening of medical images. In the current hospital system, the PET-CT scans generate about 640 images per patient. If the image is processed and screened in a manual form, a large amount of labor, material, and human resources will be wasted. If all images are stored and processed, 640 images need about 2 M bytes of space, and only part of the 640 images can be used as effective processing information, which will result in the waste of medical system resources. The other is to calculate the concentration value of the prostate cancer lesion area and carry out edge detection and processing on the effective images to obtain the corresponding lesion area, which can be used as the input information of prostate cancer machine-assisted diagnosis system under the big data environment.

The second stage is the input model of the machine-assisted diagnosis system for prostate cancer under the big data environment. All the obtained digital information will be preprocessed properly, and the irregular, unreasonable, and invalid information is transformed into qualified and effective digital information. Six different disease indexes (tumor markers) can be used to evaluate the clinical staging of prostate cancer produced by two kinds of medical image indexes: the lesion area and lesion area density; then, these two kinds of data integration generate input vectorACP1andACP2, to the different data models.

The third stage is the neural network model of the machine-assisted prostate cancer diagnosis system under the big data environment, which is the operation process of the input layer, hidden layer, and output layer of the neural network. The whole neural network model combined with the diagnosis process of prostate cancer is divided into two parts. The first part is a large number of prostate cancer cases collected and counted in the early stage as the data information of model training, with the purpose of constantly adjusting the weight and bias between various points. Positron emission computed tomography (PET) and computed tomography (CT) patients with six different disease indicators (tumor markers): Hb, PSMA, FPSA, RBC, PAP and TPSA, and two medical image indicators. The lesion area is the density of the lesion area generated by the two vectors as the input of the input layer. The disease index and the medical image are finally found to be the return error of the two-part model parameter adjustment.

The fourth stage is the decision-making model of the machine-aided diagnosis system for prostate cancer under the environment of big data. The final output value is obtained by combining the prostate cancer disease index and the output of the prostate cancer medical image. Combine two different output values, that is, combine the prostate cancer disease index and the prostate cancer medical imaging index to adjust the weight. Because the clinical diagnosis of prostate cancer mainly relies on serum PSA, when digital rectal examination through rectal ultrasound and pelvic MRI are performed through digital rectal examination, CT is less sensitive to the diagnosis of early prostate cancer. Therefore, the weight of the image in the early diagnosis of prostate cancer should be less than that in the later stage. This requires a corresponding algorithm to combine the two, and finally draw a DPC system output. Then according to the final output value (DPC), the cancer stage is assisted in diagnosis. According to the Tumor Node Metastasis (TNM) guidelines, prostate cancer can be divided into four stages (I, II, III, and IV): prophase, early stage, middle stage, and late stage. The clinical stage of prostate cancer can be comprehensively evaluated according to the final combined output value (DPC).

### 2.2. The Machine-Aided Diagnosis System for Prostate Cancer Inputs Medical Images for Preprocessing in the Context of Big Data

The clinical manifestations of prostate tumors are the obvious enlargement of tumor and the diffusion of water molecules in living tissues, the increase of cell density, the change of the macromolecular protein content, and the increase of cell density in malignant lesions of cancer cells, which leads to the increase of tumor plane area and darker color of medical image. Therefore, the article uses the lesion area and its gray level of medical images as indicators to assist in the diagnosis.

In the context of big data. Machine-assisted diagnosis of prostate cancer is based on six different disease indicators (tumor markers): HB, PSMA, FPSA, RBC, PAP and TPSA, as well as PET and CT. These two vectors are the lesion area and the density of the lesion area generated as input to the input layer. In terms of medical images of prostate cancer, we are not able to directly obtain the input of two image indexes through PET-CT images. Therefore, this will require preliminary preprocessing of medical images.

First of all, in some medical images, there may be some color labeling, which is called the noise of the image. Therefore, we performed gray-scale processing of prostate cancer medical images to obtain a complete gray-scale cancer image:

(1) $$\begin{array}{c} P_{\text{Gray}}=P_{R}*\alpha +P_{G}*\beta +P_{B}*\gamma . \end{array}$$

*P*_*R*_, *P*_*G*_, and *P*_*B*_, respectively, correspond to the three colors of the original RGB color pattern in the original image. According to the characteristics of prostate cancer medical images, the value range of parameter *α* is 0.25~0.35, the value range of *β* is 0.55~0.65, and the value range of *γ* is 0.1 ~ 0.2.

Our ultimate goal is to get the focus area of the medical image of prostate cancer. And because of that, the medical image has a lot of noise; we want to remove the noise of the cancer image. The purpose of Gaussian blur is mainly to reduce the overall image noise. The purpose is to calculate the image gradient and edge more accurately *P*_*Gray*_:

(2) $$\begin{array}{c} h\left(x,y,\sigma \right)=\frac{1}{2{\pi \sigma }^{2}}\exp\left(-\frac{x^{2}+y^{2}}{2\sigma^{2}}\right) \end{array}$$

where *σ* determines the Gaussian variance for the input parameter in order to determine the degree of blurring of the cancer image.

Then, Gaussian smoothing was performed on the prostate cancer image to obtain the processed prostate cancer image *f*(*x*, *y*)*g*(*x*, *y*):

(3) $$\begin{array}{c} g\left(x,y\right)=h\left(x,y,\sigma \right)*f\left(x,y\right) \end{array}$$

where ∗ represents convolution, which will be converted into a two-dimensional template for the operation of convolution on cancer images *h*(*x*, *y*, *σ*).

The gradient and amplitude of prostate cancer medical images were then calculated. Image gradient, the direction of the gradient is the direction where the function *f*(*x*, *y*) changes fastest. When there is an edge in the image, there must be a large gradient value. On the contrary, when there is a relatively smooth part in the image, the gray value changes little, and the corresponding gradient is also small. In image processing, the mode of gradient is referred to as gradient, and the image composed of image gradient is called gradient image. The edge of the image has two properties, direction and magnitude. The pixel changes gently along the edge direction and dramatically perpendicular to the edge direction. This change on the edge can be detected by the differential operator, usually using the first or second derivatives to detect edge. We calculate the amplitude and direction of the gradient with the first-order finite difference.

The gradient of the medical image of prostate cancer after smoothing and filtering can be used to calculate the partial derivatives of *x* and *y* with the first-order finite difference approximation of 2 × 2*g*(*x*, *y*)*f*_*x*_′(*x*, *y*)*f*_*y*_′(*x*, *y*):

(4) $$\begin{array}{c} \left\{\begin{array}{c} f_{x}^{\prime }\left(x,y\right)\approx G_{x}=\frac{f\left(x+1,y\right)-f\left(x,y\right)+f\left(x+1,y+1\right)-f\left(x,y+1\right)}{2},\\ f_{y}^{\prime }\left(x,y\right)\approx G_{y}=\frac{f\left(x,y+1\right)-f\left(x,y\right)+f\left(x+1,y+1\right)-f\left(x+1,y\right)}{2}. \end{array}\right. \end{array}$$

In a rectangular coordinate system, the relation between coordinates, amplitude, and azimuth is

(5) $$\begin{array}{c} \left\{\begin{array}{c} M\left(x,y\right)=\sqrt{G_{x}{\left(x,y\right)}^{2}+G_{y}{\left(x,y\right)}^{2},}\\ Q\left(x,y\right)=\arctan\left(\frac{G_{x}\left(x,y\right)}{G_{y}\left(x,y\right)}\right). \end{array}\right. \end{array}$$

For prostate medical image, there is a need to get the focus area and need to get the edge image of the focus area, where *M*(*x*, *y*) represents the amplitude, which reflects the edge intensity of the cancer image; *Q*(*x*, *y*) represents the azimuth, which reflects the direction of the gradient; therefore, when *M*(*x*, *y*) obtained the local maximum, its corresponding gradient direction *Q*(*x*, *y*) reflects the direction of the edge (the edge direction is perpendicular to the gradient direction), as shown in the figure.

In order to obtain more accurate edge images of cancer lesion areas, nonmaximal suppression of gradient amplitude is also required at last; that is, the larger the value in the current gradient amplitude matrix is, the corresponding edge point of the cancer image is at this point, which can only indicate that the gradient value of this point is larger. In this way, the step of nonmaximum suppression is completed. A large part of nonedge points are removed, and binary image output is finally obtained, as shown in the figure.

At this point, after detecting the edge of the prostate cancer lesion area, we will try to obtain one of the input parameter areas. This system uses the eight-direction Freeman chain code to calculate the irregular area.

The reason why it is called eight-direction chain code is that it starts from the starting pixel point and scans the next adjacent pixel point, then looks at the relative position of the original pixel point and the adjacent pixel point, encodes according to the eight-direction diagram, and scans successively to obtain the Freeman chain code.

Then, carry out front vector and back vector annotation on the chain code:

(6) $$\begin{array}{c} s1\left[i\right]=\left\{\begin{array}{ccccc} -1, & i>0, & pi\times 1\left[i\right]\cdot y>pi\times \left[i-1\right]\cdot y, & i=0, & pi\times 1\left[0\right]\cdot y>pi\times \left[N-1\right]\cdot y,\\ 0, & i>0, & pi\times 1\left[i\right]\cdot y=pi\times \left[i-1\right]\cdot y, & i=0, & pi\times 1\left[0\right]\cdot y=pi\times \left[N-1\right]\cdot y,\\ 1, & i>0, & pi\times 1\left[i\right]\cdot y<pi\times \left[i-1\right]\cdot y, & i=0, & pi\times 1\left[0\right]\cdot y<pi\times \left[N-1\right]\cdot y, \end{array}\right\}\\ s2\left[i\right]=\left\{\begin{array}{ccccc} -1, & i<N-1, & pi\times 1\left[i+1\right]\cdot y>pi\times \left[i\right]\cdot y, & i=N-1, & pi\times 1\left[N-1\right]\cdot y>pi\times \left[0\right]\cdot y,\\ 0, & i<N-1, & pi\times 1\left[i+1\right]\cdot y=pi\times \left[i\right]\cdot y, & i=N-1, & pi\times 1\left[N-1\right]\cdot y=pi\times \left[0\right]\cdot y,\\ 1, & i<N-1, & pi\times 1\left[i+1\right]\cdot y<pi\times \left[i\right]\cdot y, & i=N-1, & pi\times 1\left[N-1\right]\cdot y<pi\times \left[0\right]\cdot y. \end{array}\right\} \end{array}$$

Add the former vector annotation and the latter vector annotation, and the result is *s*[*i*]:

(7) $$\begin{array}{c} s\left[i\right]=\left\{\begin{array}{c} -1,\\ 0,\\ 1, \end{array}\right.\;\begin{array}{c} \text{a}=1,2,3,\\ \text{a}=0,4,\\ \text{a}=5,6,7. \end{array} \end{array}$$

The final target area can be expressed as

(8) $$\begin{array}{c} P_{\text{Area}}=\overset{N-1}{\underset{i=0}{\sum }}pix1\left[i\right]x*s\left[i\right]+N_{1}. \end{array}$$

After converting the original image to grayscale image, calculate another parameter of the model input:

(9) $$\begin{array}{c} P_{\text{Gray}‐\text{level}}=\frac{1}{\text{nl}}\overset{\text{nl}}{\underset{i=1}{\sum }}V_{\text{pi}}, \end{array}$$

where nl is the total pixel point in the prostate cancer lesion area and is the pixel value corresponding to the pixel point *V*_pi_.

In this module, we have two purposes: first, the model needs to screen the medical images of prostate cancer through two indicators; second, the need to finally obtain the input and *P*_Area_*P*_Gray‐level_.

### 2.3. Input Model of the Machine-Aided Diagnosis System for Prostate Cancer in the Context of Big Data

The input model of the machine-aided diagnosis system of prostate cancer in the context of big data needs to properly process all the digital information obtained and convert irregular, unreasonable, and invalid information into qualified and effective digital information. In particular, such digital information cannot be faulted, and it is more necessary to process irregular and unreasonable invalid information into effective input, so as not to affect the operation of the system.

In the prostate cancer machine-assisted diagnosis system in the context of big data, the input is divided into two parts. One part is the input of disease indicators into ACP(*t*). Among these test results, tumor markers are the most valuable signals for diagnosis, treatment, and prognosis. Therefore, the prostate disease index input consists of six disease indexes, namely, HB, PSMA, FPSA, RBC, PAP, and TPSA. The vector ACP(*t*) that combines these six disease indexes is the input to the system:

(10) $$\begin{array}{c} \text{ACP}\left(t\right)=\left[X_{\text{TPSA}},X_{\text{RBC}},X_{\text{HB}},X_{\text{FPSA}},X_{\text{PAP}},X_{\text{PSMA}}\right]. \end{array}$$

Although the disease index of prostate cancer is an important indicator to assist the diagnosis, it is obviously too superficial and insufficient to simply rely on the disease index as the judgment and decision. Therefore, based on taking the disease index as the input of the system to make the diagnosis decision, the system also adds a part of the input image index input AHP(*t*). AHP(*t*) is two kinds of medical image indexes, the lesion area and lesion area density, produced by PET-CT taken by patients. And these two indicators form a vector AHP(*t*) as another input to the system *P*_Area_*P*_Gray‐level_:

(11) $$\begin{array}{c} \text{ACP}\left(t\right)=\left[P_{\text{Area}},P_{\text{Gray}‐\text{level}}\right]. \end{array}$$

The input model is not only the beginning of the theoretical machine-aided diagnosis system but also the beginning of the two input neural network models in the input layer of neural network.

### 2.4. Neural Network Model of Machine-Aided Diagnosis System for Prostate Cancer under Big Data Environment

This part is the study of prostate cancer by six different disease indexes (tumor markers), Hb, PSMA, FPSA, RBC, PAP, TPSA, and PET-CT of two kinds of medical image metrics, the lesion area *P*_Area_ and lesion area density *P*_Gray‐level_ generated by the two vectors as input, and then adjusted by weight to study the diagnosis process of prostate cancer by disease index and medical image, respectively. Therefore, this stage of research should be divided into two parts: the first part is the reasonable evaluation and allocation of the weight of disease indicators and medical image indicators; the second part is the diagnosis process of the intelligent diagnosis system, and a preliminary diagnosis result is obtained by adjusting the weight and bias of tumor markers.

#### 2.4.1. Reasonable Evaluation and Allocation of Disease Index and Image Index Weight of Prostate Cancer

In this study, the inpatient information of more than 8,000 prostate patients in three hospitals in China was studied, and about 2,156,528 data items were collected and statistically analyzed. Therefore, under such a large data volume, we can continuously adjust and evaluate the weight through experiments. The neural network model of the machine-aided diagnosis system of prostate cancer in the big data environment is a process of reasonably adjusting the weight and deviation of each node of prostate cancer after input. According to the overall structure of intelligent medical big data decision model diagnosis system, the neural network model following the process of the diagnosis of prostate cancer is divided into two parts. On the left side is through early collection of statistics of a large number of cases of prostate cancer as a model of training data information; the goal is to continuously adjust the weights and bias between the various points. With six different disease indexes (tumor markers): Hb, PSMA, FPSA, RBC, PAP, and TPSA generated by the vector ACP(*t*) as input; intelligent diagnosis system is ultimately getting the return error of disease index to adjust the parameters of the intelligent diagnosis system. The right part is a large number of prostate cancer case images collected and counted in the early stage, and two medical image indexes obtained through preliminary preprocessing are used as data information of model training, so as to constantly adjust the weight and bias between various points. And in patients with a type of PET-CT of two kinds of medical image indexes, the lesion area and lesion area density, generated vector AHP(*t*) as input, intelligent diagnosis system is ultimately getting the return error of disease index of intelligent diagnosis system with parameter adjustment *P*_Area_*P*_Gray‐level_. Therefore, the input of the two parts can be expressed by vectors as follows:

(12) $$\begin{array}{c} \left\{X_{\text{TPSA}},X_{\text{RBC}},X_{\text{HB}},X_{\text{FPSA}},X_{\text{PAP}},X_{\text{PSMA}}\right\}=\text{ACP}\left(t\right),\\ \left\{X_{\text{Area}},X_{\text{Gray}‐\text{level}}\right\}=\text{AHP}\left(t\right). \end{array}$$

When the ACP(*t*) and AHP(*t*) generated by six tumor markers and two medical image indicators are inputted into the intelligent diagnosis system, the weight between neurons is adjusted to form the input of the next neuron, and each neuron has a corresponding activation function, which is used to process the data.

The activation function selected is sigmoid function, which is a common S-shaped function in biology, also known as the S-type growth curve. Because its value range is (0, 1), the input vector ACP(*t*) and AHP(*t*) of prostate cancer can be mapped to the interval (0, 1). In the process of big data medical-aided diagnosis of prostate cancer, there is no linear connection between the upper and lower layers of the neural network, and the difference between these features is complex but not large. Therefore, sigmoid function is a suitable choice for the model of big data-assisted diagnosis of prostate cancer.

(13) $$\begin{array}{c} f=\frac{1}{1+e^{-x}} \end{array}$$

In a multilayer network, the output of the previous layer will serve as the input of the later layer:

a^*m*+1^_(ACP(*t*), AHP(*t*))_ = *f*^*m*+1^(*W*^*m*+1^a^*m*^ + *b*^*m*+1^),  *m* = 0, 1, ⋯⋯, *M* − 1. (16).

*M* is the number of layers in the neural network. Six disease indicators and two medical image indicators can be calculated by weight to obtain a final cancer result, which we call the expected result (the desired result). Comparing the expected result and the actual result (actual results), the error function will reach the minimum value min*E* according to the gradient descent method. The purpose is to make the diagnosis decision result more and more close to the real value by adjusting the weight, so that the weight and bias can reach a state of “perfect”. The error function of desired result and actual results are

(14) $$\begin{array}{c} E_{\left(\text{ACP}\left(t\right),\text{AHP}\left(t\right)\right)}\left(w,b\right)=\frac{1}{2}\overset{n-1}{\underset{k=0}{\sum }}{\left(D_{\text{Desired result}}-D_{\text{actual results}}\right)}^{2}. \end{array}$$

Among them, the number of nodes*n*as the final output to diagnose prostate cancer under the large data of expected results with the real result would be a differentiation value on prostate cancer; we eventually have to reach the goal to get the value to minimize the differentiation of prostate cancer. When it reaches a certain small value is an optimal solution, and finally, we, through the optimal solution to adjust the weights and bias, optimize weights and bias:

(15) $$\begin{array}{c} w_{\left(\text{ACP}\left(t\right),\text{AHP}\left(t\right)\right)}=w_{\left(\text{ACP}\left(t\right),\text{AHP}\left(t\right)\right)}-\eta_{1}\frac{\partial E\left(w,b\right)}{\partial w_{\left(\text{ACP}\left(t\right),\text{AHP}\left(t\right)\right)}}=w_{\left(\text{ACP}\left(t\right),\text{AHP}\left(t\right)\right)}-\eta_{1}\delta x,\\ b_{\left(\text{ACP}\left(t\right),\text{AHP}\left(t\right)\right)}=b_{\left(\text{ACP}\left(t\right),\text{AHP}\left(t\right)\right)}-\eta_{2}\frac{\partial E\left(w,b\right)}{\partial b_{\left(\text{ACP}\left(t\right),\text{AHP}\left(t\right)\right)}}=b_{\left(\text{ACP}\left(t\right),\text{AHP}\left(t\right)\right)}-\eta_{2}\delta , \end{array}$$

*η* is learning efficiency, and diagnostic accuracy is the essential requirements of the whole model. The higher the accuracy is, the more perfect the intelligent diagnosis system is. We adjust the weight; the diagnosis accuracy is the purpose of increase through six tumor markers and two medical image indexes which generatedACP(*t*)andAHP(*t*)two inputs; through the distribution of the weight, get the final diagnosis decision results. Therefore, it is crucial to adjust the weights and bias to achieve a “perfect state”.

#### 2.4.2. The Diagnosis Process of Prostate Cancer Intelligent Diagnosis System

In the first stage, we have obtained the weight and bias between each node by training with a large amount of data. That is to say, the ownership weight of the model of big data intelligent medical auxiliary diagnostic system has been determined. Therefore, at this stage, the process of the diagnosis of intelligent system will be analyzed. First of all, the six different disease indexes (tumor markers): HB, PSMA, FPSA, RBC, PAP, TPSA, and PET-CT of two kinds of medical image metrics: two vectors ACP(*t*) and AHP(*t*)generated by Area and Gray-level are used as inputs.

The next section will introduce the relationship between disease indicators and image indicators and how to adjust the weight proportion between the comprehensive two diagnosis results.

### 2.5. Decision-Making Model of Machine-Aided Diagnosis System for Prostate Cancer in the Context of Big Data

#### 2.5.1. Decision Model of Expert System Based on Rule Reasoning

From the previous step, the article has obtained the auxiliary diagnosis results derived from prostate disease indicators and medical imaging indicators. But the final decision needs to combine the two to make a decision diagnosis of prostate cancer. First, you need to obtain a comprehensive index and then make a decision diagnosis, so that the decision can be more accurate and reliable. Therefore, this section introduces the relationship between the disease index and the image index and how to adjust the weight ratio between the two comprehensive diagnosis results.

Among the decision models, the rule reasoning model is a new weight adjustment algorithm introduced by us, which is developed from the rule-based expert system, a branch of the expert system in the field of artificial intelligence. In the decision-making model of the machine-aided diagnosis system of prostate cancer in the context of big data, the article uses a simpler expert system hair model based on rule reasoning.

**Figure 2:**
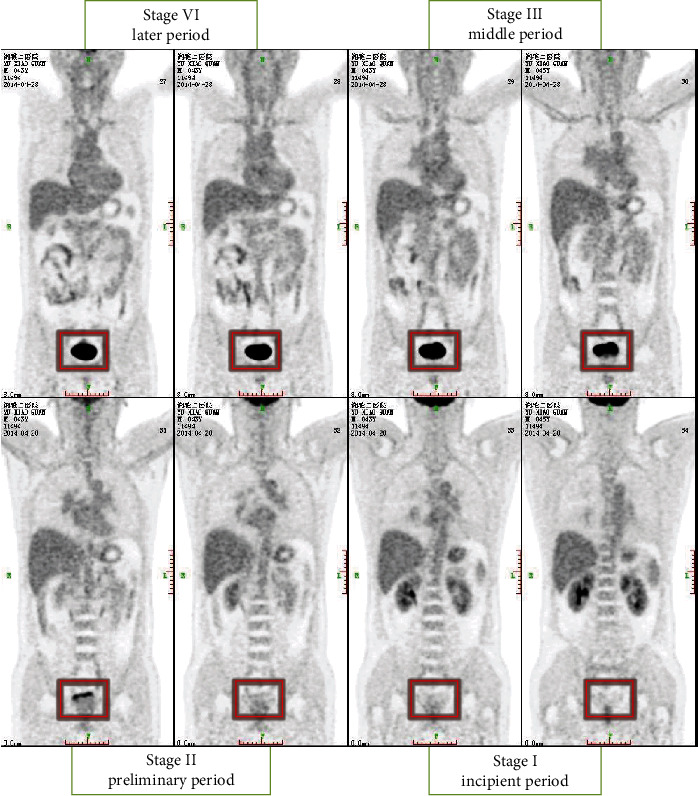
The clinical staging marker image of prostate cancer.

The rule-based inference model is a knowledge set expressed by rules, including the knowledge needed for reasoning execution. As shown in Figure 2, in the process of the diagnosis of prostate cancer, low-level prostate cancer clinical symptoms are not particularly obvious, but in a high level of clinical symptoms of prostate cancer is obvious and in the condition of prostate cancer research found that when patients clinical staging of prostate cancer in stage III or IV stage image characteristics than I or II when more obvious, therefore, if the step we have got prostate disease index and auxiliary diagnosis of medical image index to are still in the stage I or II, this may cause the index weight of the disease to be greater than the image index.

In the working memory of the rule-based inference model, the complete data set required for inference execution will be obtained. And the analysis results can be roughly obtained by analyzing the changes of the index characteristics corresponding to different clinical stages as shown in Table 1.

Finally, it is the setting of an inference engine. When two diagnostic results are taken as conditions, in order to complete the inference process, it is necessary to determine which rule in the current cycle needs to be activated. In the decision-making model of the machine-assisted prostate cancer diagnosis system in the context of big data, each diagnosis is a definite value, and each set of conditions has a corresponding data in the workspace. Therefore, in the decision-making model of the machine-aided diagnosis system of prostate cancer in the context of big data, when conditions are determined, the corresponding rules can be determined, and usually there will be no rule conflict.

#### 2.5.2. Staging Management and Treatment Suggestions of Prostate Cancer Machine-Assisted Diagnosis System under Big Data Environment

In the decision-making model of the machine-assisted prostate cancer diagnosis system in the context of big data, get the final result through rule-based reasoning model DPC. Based on this result, perform cancer staging and then cancer machine-assisted diagnosis staging based on the final DPC. According to the tumor node metastasis (TNM) guidelines, prostate cancer is mainly divided into four stages (I, II, III, and IV) in the early, middle, and late. Based on the final output value (DPC), the clinical staging of prostate cancer was comprehensively evaluated.  Table 2 shows the staging threshold of the machine-assisted diagnosis system for prostate cancer. At the same time, through the comprehensive evaluation of clinical staging judgment and phase estimation, the machine-aided system can recommend different treatment schemes to doctors, such as active monitoring, drug therapy, resection, endocrine therapy, radiotherapy, and chemotherapy.

## 3. Experimental Performance

### 3.1. Experimental Data Collection, Classification, and Preprocessing

**Figure 3:**
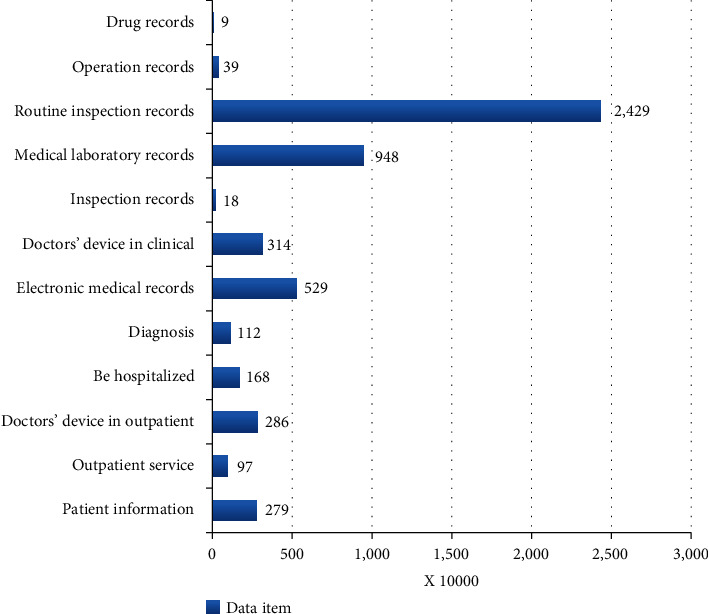
The classification of items for collecting medical data for prostate cancer patients.

1. Before the experiment, collect all the medical information needed from three first-class hospitals in China: Xiangya Hospital, Xiangya Second Hospital, and Xiangya Third Hospital, and classify these information. Besides, the data center collects various information items from different aspects to help the system-assisted diagnosis
2. The team collects a large number of prostate cancer medical data and makes corresponding classification statistics and preprocessing and then does the corresponding recording work. Because of the large amount of medical data collected, although it is medical data related to prostate cancer, only a part of the medical data is used in this system. Therefore, in order to ensure the smooth progress of the experiment and its rationality and accuracy, we extracted more than 8,000 prostate patients' data from the data collected by the three hospitals and extracted 16,143 items of systematic structured and effective information from the data of these patients.  Figure 3 shows the classification items of medical data information about prostate cancer collected by the three hospitals

### 3.2. Initialize System Data Variables

(1) After testing and analyzing more than 8,000 cases of prostate patients as a training set, some parameters of the intelligent medical system are set as follows: initial weight determination; initial weight should be set to different values. Therefore, as for the initialization of the weights, design a random occurrence program to generate a set of 0 ~ 0.5 random numbers as the initial weight of the network; as for the minimum training rate, the initial value can be set to 0.9 since the training rate will be automatically adjusted; as for the dynamic coefficient, its selection is generally 0.6 ~ 0.8; the allowable error is 0.001~0.00001. According to clinical medical standards, some standard medical indicators data are shown in Table 3.

(2) The intelligent system image index initialization information is preprocessed according to two medical image indicators generated by PET-CT, and the area of the lesion area and the lesion area are obtained. Gray-level two image indicators, the resulting vectorACP(*t*), as the initial input to the intelligent system.

**Figure 4:**
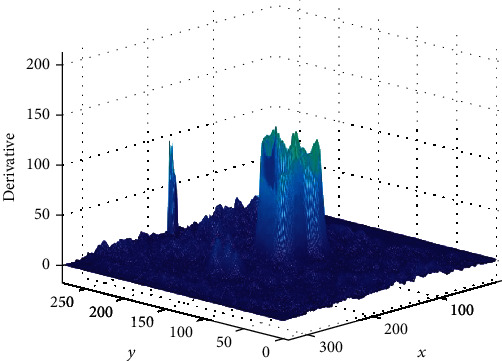
Three-dimensional image of pixel intensity in prostate cancer lesions.

**Figure 5:**
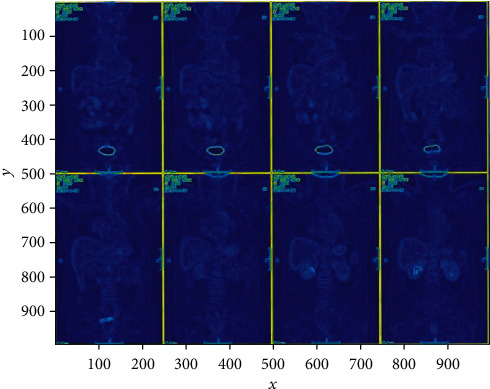
Plane display of pixel intensity in prostate cancer lesions.

In this process, because prostate cancer images have a lot of noise, preexperiment experiments were carried out through a large number of images of prostate cancer before the experiment, and various parameters suitable for processing prostate cancer images were calculated and grayed out by filters and double thresholds. The postprocessing of the image is shown in Figure 1. The edge intensity of the lesion area is compared with the surrounding edge intensity. The resulting edge range will be more accurate and ensure that the final intelligent system correctly diagnoses the diagnosis. Figures 4 and 5 show the comparison of the intensity of edge pixels after detection with the intensity of surrounding pixels. It can be clearly seen that the edge intensity is much higher than the surrounding pixel intensity. In Figure 5, the *x*-axis and *y*-axis is the length and width of the image, respectively, and the *z*-axis corresponds to the gray level of the grayscale image. The 3D effect shows that the gray scale of the prostate cancer lesion area is much higher than that of other regions.

**Figure 6:**
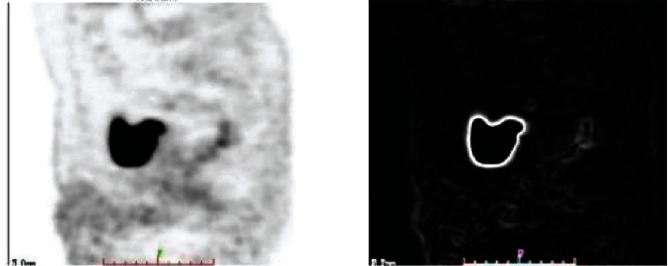
Comparison of grayscale image and gradient amplitude image.

**Figure 7:**
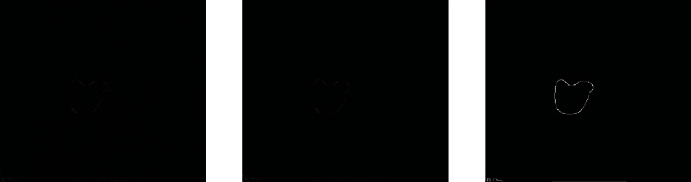
Nonmaximum suppression image and binary image output.

The whole preprocessing process is to first perform grayscale processing on the image and then calculate the gradient amplitude of the grayscale image, nonmaximum value suppression, and the threshold processing finally obtains the binary image output and obtains a binary value suitable for calculating the area of the lesion area. The image is calculated by the Freeman chain code for the irregular connected graph, as shown in Figures 6 and 7:

After obtaining the two-part data index, the system uses the data index generation vector as the initial input of the system model. Only accurate indicator data can make an accurate auxiliary diagnosis.

**Figure 8:**
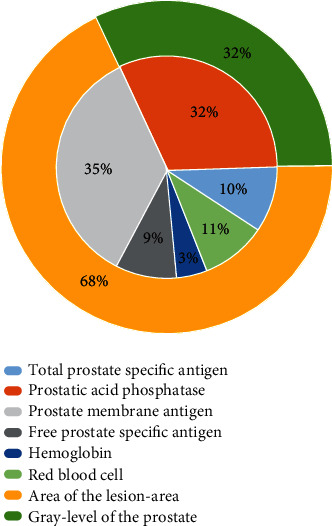
Weight distribution of various indicators of prostate cancer.

(3) In the big data environment of more than 8,000 patients with prostate cancer, the intelligent medical system trained the data to get the corresponding weight. The intelligent system has a specific numerical division of the four clinical stages of prostate cancer. In terms of medical disease indicators, the smart medical system is through six prostate cancer disease indicators and two medical image indicators by patients were used for assisted diagnosis. Therefore, in the two modules of prostate cancer disease indicators and medical image indicators, according to the weight of each disease indicator and medical image indicators plus the training result data, the system will give the weight between every two nodes reasonable. The weight distribution of prostate cancer disease indicators and image indicators is shown in Figure 8.

(4) In the big data intelligent medical system decision model, in order to accurately determine the clinical stage of prostate cancer, the system combines the disease index and the medical image index to assist the diagnosis. However, prostate cancer often occurs in the posterior lobe, and there are no obvious symptoms in the early stage. Patients with prostate cancer often have symptoms such as frequent urination, difficulty in urinating, thinning of urine, prolonged urinary tract, dysuria, and urinary retention. Prostatic hyperplasia is the same, so it is difficult to diagnose prostate cancer based on this, mainly relying on digital rectal examination to make judgments. Therefore, in the rule-based reasoning algorithm, set the rules to the early, middle, and late three weights. Because medical images are not very obvious in early prostate cancer, if the prostate cancer disease index model and the image index model result are in the early stage, the image model will occupy a smaller proportion. If the prostate cancer index model and the image index model results are at a later stage, the proportion of medical image indicators is gradually increasing.  Table 4 shows the rational distribution of the corresponding weight *W*_disease_indicator_ of the disease indicator. The corresponding medical image indicator is expressed as *W*_image_indicator_:

(16) $$\begin{array}{c} W_{\text{image}\_\text{indicator}}=1-W_{\text{disease}\_\text{indicator}}. \end{array}$$

As shown in Table 4, since medical imaging has no obvious characteristics in early prostate cancer, the reasonable allocation weights of disease indexes and imaging indexes are divided into 4 cases to set the weights reasonably. (a) When both the prostate disease index and the medical imaging index point to stages I and II, the weight of the disease index is set to be slightly larger than the weight of the medical imaging index. (b) When both the prostate disease index and the medical imaging index point to the third and fourth periods, the weight of the disease index is set to be slightly smaller than the weight of the medical imaging index. The other two situations are somewhat special. The clinical stage of the disease index model and the medical image model are completely different. Because in the early stages of prostate cancer, medical image detection is indeed very convincing. Therefore, in order to ensure accuracy and rationality, our weight design has been processed as follows. (c) When the medical image index points to stages I and II and the prostate disease index points to stages III and IV, the weight of the disease index is set to be much larger than the weight of the image index. (d) When the medical image index points to stages III and IV, and the prostate disease index points to stages I and II, the weight of the disease index is set to be much smaller than the weight of the image index.

### 3.3. Data Evaluation and Analysis of Prostate Cancer in the Big Data Environment

**Figure 9:**
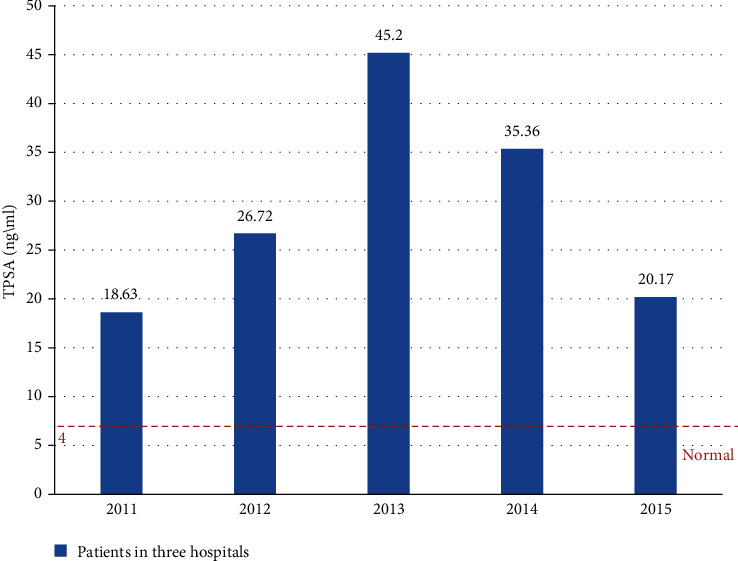
The TPSA growth trend of the three hospitals from 2011 to 2015.

TPSA is a specific marker of prostate cancer, and it is also meaningful for the diagnosis of prostate cancer with no obvious symptoms in the early stage. As shown in Figure 9, in the five years from 2011 to 2015, the average value of TPSA from 2011 to 2013 increased from 18.63 ng/ml to 45.2 ng/ml, reaching its peak in 2013, but declined from 2013 to 2015. The trend in the past five years indicates that the condition of prostate patients is effectively controlled. It is generally believed that TPSA is less than 4 ng/ml normal value, and TPSA is greater than 10 ng/ml, which indicates an increased risk for prostate cancer patients. When the average level exceeds 50 ng/ml, the patient is likely to have prostate cancer.

**Figure 10:**
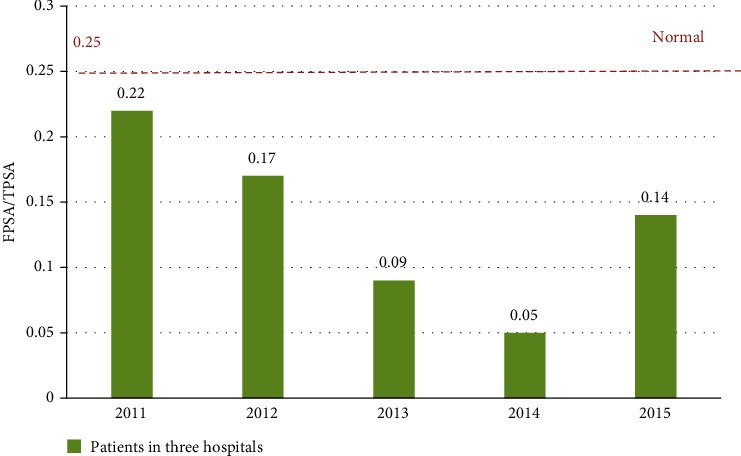
The changes in FPSA/TPSA trends in the three hospitals from 2011 to 2015.

FSPA/TSPA, another important diagnostic indicator for the big data intelligent assisted diagnostic system, is also an important basis for assisted diagnosis and treatment. As shown in Figure 10, the normal range of FPSA/TPSA is around 0.25, but for the five years from 2011 to 2015, the FPSA/TPSA values are below 0.25 and are in a decline from 2011 to 2014. The value of FPSA/TPSA has fallen to 0.05 in 2014, and when FPSA/TPSA is below 0.1, the incidence of cancer will reach a high level of 56%. This shows that most patients with prostate cancer have been deteriorating during this period time and are not well-controlled. In these structured medical data, we can further realize that in these five years, most of the patients in the three hospitals are in the stages III and IV of cancer.

So far, the causes of prostate cancer have been divided into two major modules, one of which is genetically leading to prostate cancer. To prove this claim, we extracted data from all major prostate patients from 2011 to 2015 and made the analysis for a dangerous proportion of data, setting the number of uninherited patients to 1, and the genetic patients are the multiples of uninherited patients. From the tens of thousands of data, we can roughly draw two conclusions. One is that the number of patients with genetic disease is more than five times of the total number of patients, which indicates that heredity is one of the major causes of prostate cancer; another point is that it can show from the trend of data that this multiple relationship is constantly rising between 2011 and 2015, and the proportion of genetic diseases is increasing at the same time as the number of patients increases. This shows that the number of genetic patients is increasing year by year.

The other is diet that leads to prostate cancer. First, people who love smoking have a higher chance of developing cancer. Many people know that smoke contains a lot of dirty things, and in the process of smoking, those substances will be sucked into the body. Second, vitamin A is a trace element required by the human body. Absorption of a certain amount of vitamin A can promote the healthy development of human body, and excessive amount will lead to a large increase in saturated fatty acids in the human body, thereby causing lesions in prostate cells. Third, carotene has a certain promoting effect on the pathogenesis of prostate cancer. Fourth, a high-fat diet is also a cause of prostate cancer because it contains many animal fats and some fatty acids. If people eat more of these things, some of the meat in the body is digested into different types of fatty acids; so in the long run, the chance of developing prostate cancer will be greatly improved. So this is why many doctors say that patients who have cancer have to eat less meat, because eating more meat will hurt their body's immune system.

**Figure 11:**
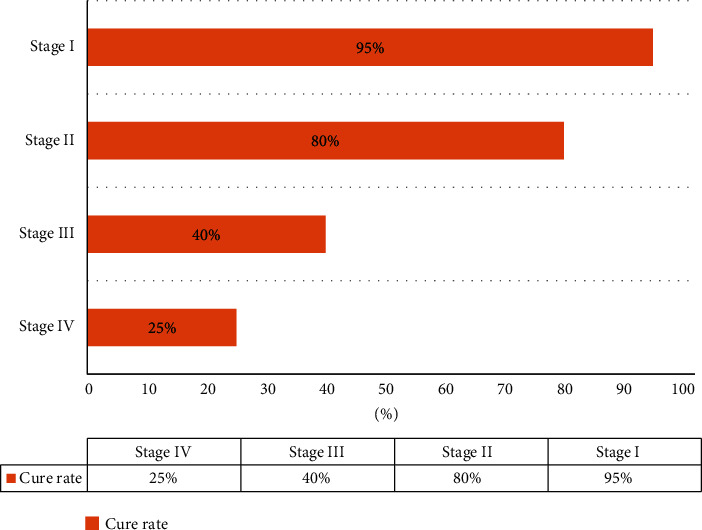
The cure rate of four stages of prostate cancer.

The incidence and mortality of prostate cancer are second only to lung cancer and ranks second in cancer mortality. As shown in Figure 11, the first-stage cure rate of prostate cancer is over 95%, the second-stage cure rate is over 80%, the third-stage cure rate is less than 40%, and the fourth-stage cure rate is only 25%. The prostate cancer intelligent diagnosis system provides patients with corresponding treatment recommendations based on the system diagnosis results, as shown in Table 5:

(17) $$\begin{array}{c} \text{True positive rate}=\frac{\text{true positive}}{\text{true positive}+\text{false negative}},\\ \text{False positive rate}=\frac{\text{false positive}}{\text{false positive}+\text{true negative}}. \end{array}$$

**Figure 12:**
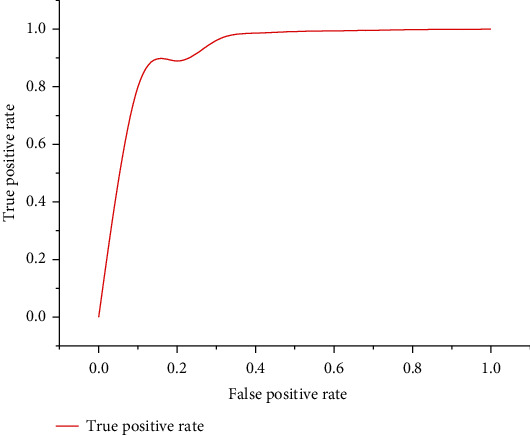
Receiver operating curve (ROC) for the classification rate of machine-assisted diagnosis of prostate cancer.

**Figure 13:**
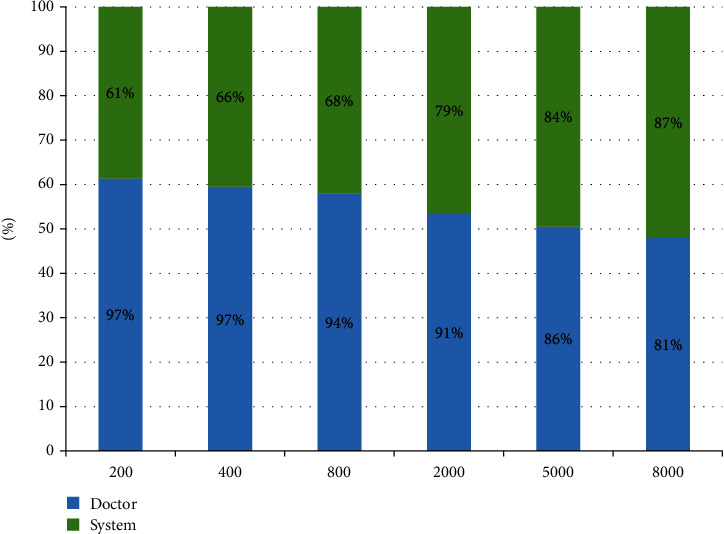
Comparison of diagnostic accuracy between doctors and machine-assisted systems.

The prostate cancer machine-assisted diagnosis system proved feasible in the experiment. As shown in Table 6 and Figure 12, the receiver operating curve (ROC), the ROC is thetrue positive rate–false positive rate curve: the*x*-axis represents true; positive rate, the*y*-axis represents the false positive rate. From Figure 12, we can see that the curve is very close to (0, 1) point [22–24]. Therefore, the classification accuracy of the prostate cancer machine-assisted diagnosis system is still very high. We can compare this accuracy with the expert doctor. The results are as follows:

The main role of the prostate cancer intelligent diagnosis system is self-evident, mainly for the preliminary diagnosis for prostate patients, and to collect statistical information according to the data. During the past five years, the vast majority of the more than 8,000 patients in the three hospitals have gradually recovered under the treatment of doctors. In this experiment, the patient enters the hospital for diagnosis, and the doctor will generate a diagnosis book for the first diagnosis. Our data are from three famous Xiangya hospitals of Central South University. The accuracy of the diagnosis is defined as the accuracy of the first patient diagnosis, not the accuracy of the patient after multiple diagnoses.  Figure 13 shows the comparison of the diagnostic accuracy between doctors and intelligent systems. The blue bar represents the doctor's diagnostic accuracy, and the green bar represents the machine-assisted diagnostic accuracy. When the number of cases is relatively low, it is obvious that the diagnosis accuracy of artificial experts is much higher than that of intelligent systems. However, as the number of cases increases, the diagnostic accuracy of artificial experts declines, while that of intelligent systems increases. According to the specific data, when the number of cases increased from 200 to 8000, the accuracy of manual expert diagnosis decreased from 97% to 81%, while the diagnostic accuracy of intelligent systems increased from 61% to 87%. Therefore, it can be concluded that when the diagnostic data reaches a certain amount, the accuracy of the intelligent diagnosis system for prostate cancer will exceed that of the artificial expert. Therefore, the intelligent auxiliary diagnosis system can assist the artificial expert as an auxiliary diagnosis.

## 4. Discussion

In recent years, more and more people have proposed the concept of intelligent medical system. Many medical systems have implemented different methods and techniques and involve many related technologies such as big data medical treatment and deep learning. Models for assisting prediction, diagnosis, and treatment have also become hot topics in medical applications and intelligence. Next, the article will introduce the research status related to our works in detail.

In the social discussion of the smart medical system, many people in the smart medical industry still hold an optimistic attitude and believe that the smart medical market is greatly guided by policies. With the development of society, smart medical will inevitably become a major trend. The medical reform is now divided into three stages: the first is digitization, which is to digitize a lot of information including medical records, medical history, and information for detecting a certain disease; the second stage is the hospitalization of mobile, this stage is more advanced than digital on the first floor, the change brought about is that the original patient and caregiver's space inside the hospital is not limited; the third stage is remoteization.

In fact, the three stages of medical intelligence are all related to the development of technology, and the reliability of technology makes medical institutions to trust enough. For example, electronic medical records are the development of smart terminals and networks in the hospital, while mobile hospitals rely on the progress of Wifi technology, and telemedicine relies on the development of 3G and 4G networks and mobile Internet and involves semiconductor manufacturing processes. Technology allows devices to work better with lower efficiency, such as the rapid transmission of image information after detection and the development of the Internet of Things, making the connections between devices and devices and people more smooth.

In recent years, the overall development of smart medical systems has been slow. The main reason lies in two points. The first point is that big data processing does not have an overall architecture. The second point is that the medical data model is messy and untargeted.

In the study of the article, the system will be able to directly connect to the CDR data interface in the hospital and batch process the medical big data. Among them, internal operations related to premedical data processing, data cleaning, and other operator operations are involved. For medical big data processing architecture design, we will use Hadoop and HDFS big data processing framework platforms, based on operator operations and SQL layer processing. In the process of concurrent processing, part of the model processing can be performed in parallel, thereby reducing a lot of time costs.

### 4.1. Research on Cancer Based on Neural Network

At present, there are more and more researches on cancer diagnosis based on neural network methods. However, the current neural network-based decision-making methods are relatively simple, basically based on disease indicators or according to medical image indicators to do auxiliary diagnosis and lack of sufficient medical data information. The weight of the decision-making stage is unreasonable. Georgia et al. proposed a convolutional neural network- (CNN-) based intelligent diagnosis system for breast cancer diagnosis and related processing, through the related image processing, with images as input, diagnose breast cancer, and classify breast cancer histopathology images [25–27].

Besides, Zhang et al. proposed an artificial neural network for the selection and accurate diagnosis of miRNA biomarkers for colorectal cancer [28] and proposed an artificial neural network (ANN) model. The designed ANN model can accurately classify sample data as cancerous or noncancerous. By using ANN, circulating miRNAs can be used as noninvasive, sensitive, and specific diagnostic markers. Li et al. [29] proposed an expert level for lung cancer detection and classification using the method of deep convolutional neural networks. A three-dimensional convolutional neural network (CNN) was designed to detect pulmonary nodules and classify them as malignant or benign diseases according to pathology and laboratory-confirmed results.

### 4.2. Research on Cancer Based on Machine Learning

Disease diagnosis tests based on deep learning models are also widely used [30]. The DenseNet neural network model was proposed to classify benign and malignant mammography images, improve the DenseNet neural network model, and invent a new DenseNet-II neural network model. In Wojciech et al., to predict clinical outcomes by analyzing time-series CT images of patients with locally advanced non-small-cell lung cancer (NSCLC), a deep learning prediction of lung cancer treatment response to a series of medical images was proposed [31].

In addition, Jung-Hoon et al. proposed a new machine learning method for early detection of hepatocellular carcinoma to help doctors solve clinical problems by combining genetic algorithms, support vector machines and feature optimization [32]. Geeitha et al. and Thangamani et al. proposed a support vector machine risk scoring system for ovarian cancer patients [33, 34]. By selecting miRNA sets, support vector machine (SVM) classifiers were constructed to analyze miRNA and clinical factors independently related to prediction, and a risk scoring system was constructed.

### 4.3. Research on Cancer Based on Fuzzy Reasoning

The diagnosis and probabilistic decision-making problem based on non-small-cell carcinoma (NSCLC) medical system has also become a hot issue. Wu et al. divided the evolution process of disease diagnosis parameters by clinical research and model movement of non-small-cell carcinoma and then established parameters and evaluated the selection stage of non-small-cell lung cancer [35]. Finally, combined with clinical data statistical analysis and probability, the system can provide accurate, rapid clinical data analysis and decision-making recommendations. In another study, [36] proposed a clustering range model based on image recognition and verified the accuracy of treatment by establishing a predictive decision algorithm.

Literature [37] introduced a new type of prostate cancer auxiliary diagnosis model based on fuzzy inference system, combined with statistical analysis of intelligent assisted diagnosis system and big data medical data decision, and finally combined with doctors' diagnosis to provide prostate cancer patients with disease diagnosis and the corresponding treatment plans. In addition, Liu et al. also proposed a breast cancer detection model based on fuzzy reasoning technology, which can determine whether the tumor classification is benign or malignant, and developed a two-layer, high-success rate classifier based on type-2 fuzzy reasoning that combines expert doctors' opinions to classify tumors in the BI-RADS category as benign or malignant [38]. Amin et al. proposed a model that was also based on fuzzy reasoning [22]. However, this study was based on a fuzzy reasoning model based on the change of time grayscale combined with the texture information of cervical images to classify patients with the risk of cervical intraepithelial neoplasia.

## 5. Conclusions

In this study, a machine-aided diagnosis system for prostate cancer based on perceptron neural network and big data was proposed to solve the problem of scarce medical resources caused by the large population and underdeveloped medical level in developing countries. The intelligent system takes the combination of six disease indicators and two medical image indicators as the input and continuously adjusts the weight and deviation of the neural network in the context of medical big data to form an intelligent diagnosis model based on the neural network. The model allows doctors to diagnose prostate patients with greater accuracy. Although the intelligent system can provide diagnostic information and decision-making results, it can only serve as an auxiliary diagnosis decision-making system for doctors and cannot completely replace doctors. However, it can largely reduce the work burden of doctors, improve the efficiency of hospitals, and greatly improve the accuracy of doctors' diagnostic. Through the study of hospitalization information of more than 8,000 prostate patients in three hospitals, about 2,156,528 data items were collected and compiled for experiment. Experimental data shows that when the patient base increases from 200 to 8,000, the accuracy of the machine-assisted diagnostic system will increase from 61% to 87%, and the doctor's diagnostic accuracy will drop to 81%, so the smart medical system can help doctors and medical experts make more effective treatments. From the study, it is concluded that when the patient base reaches a certain number, the diagnostic accuracy of the machine-assisted diagnosis system will exceed the doctor's expert.

In the future work, we will transfer the main work centers to big data and intelligent identification and constantly try new algorithm research, such as SVM and decision tree algorithms, or deep learning algorithms, by comparing the pros and cons between them, continuously optimizing the system model to continuously improve the accuracy of the diagnosis.

## Figures and Tables

**Table 1:** Reasonable distribution of weights corresponding to different staging results.

	Case1	Case2	Case3	Case4
Case1	Weight (C1, C1)	Weight (C1, C2)	Weight (C1, C3)	Weight (C1, C4)
Case2	Weight (C2, C1)	Weight (C2, C2)	Weight (C2, C3)	Weight (C2, C4)
Case3	Weight (C3, C1)	Weight (C3, C2)	Weight (C3, C3)	Weight (C3, C4)
Case4	Weight (C4, C1)	Weight (C4, C2)	Weight (C4, C3)	Weight (C4, C4)

**Table 2:** The staging threshold of the machine-assisted diagnosis system for prostate cancer.

Clinical stage	Stages I	Stages II	Stages III	Stages IV
Threshold (DPC)	(0, 0.25]	(0.25, 0.50]	(050, 0.75]	(0.75, 1.00]

**Table 3:** The standard indicator data for normal people.

Indicator	Normal range
Total prostate-specific antigen (TPSA)	0-4.0 ng/ml
Free prostate-specific antigen (FPSA)	4-20 *μ*g/L
Hemoglobin (HB)	120~165 g/L
Red blood cell (RBC)	12~15 g/100 ml
Acid phosphatase (PAP)	0~3.5 ng/ml
Prostate-specific membrane antigen (PSMA)	0~4 ng/ml
Area of the lesion-area	<15 mm
Gray-level of the prostate	<180

**Table 4:** Weight distribution of combining disease indicators and image indicators.

Disease indicators	Stage I	Stage II	Stage III	Stage IV
Image indicators				
Stage I	*W*_image_indicator_<*W*_disease_indicator_		*W*_image_indicator_<<*W*_disease_indicator_	
Stage II				

Stage III	*W*_image_indicator_>>*W*_disease_indicator_		*W*_image_indicator_>*W*_disease_indicator_	
Stage IV				

**Table 5:** Treatment recommendations of four stages for prostate cancer.

Clinical stage	Treatment advice
Stage I	Pay attention to physical changes and observe carefully
Stage II	Prostatectomy, radioactive implantation
Stage III	Endocrine therapy, orchiectomy (nonsteroidal antiandrogen intermittent therapy)
Stage IV	Chemotherapy (CTX, 5-FU, ADM, VLB, and PTX)

**Table 6:** Parameter-lists of true and predicted parameter for machine-assisted diagnosis of prostate cancer.

	Actual value		Total
Predictive value	True positive (*TP*)	False positive (*FP*)	*P*′
	False negative (*FN*)	True negative (*TN*)	*N*′

Total	*P*	*N*	

## Data Availability

The data used to support the findings of this study are available from the corresponding author upon request.

## Abbreviations

PCA: Prostate cancer
PET: Positron emission computed tomography
CT: Computed tomography
PAP: Prostatic acid phosphatase
PSMA: Prostate membrane antigen
HB: Hemoglobin
TPSA: Total prostate-specific antigen
RBC: Red blood cell
FPSA: Free prostate-specific antigen
MRI: Magnetic resonance imaging
TNM: Tumor node metastasis
ROC: Receiver operating curve
NSCLC: Non-small cell lung cancer.
